# Supplementary material for: Seminoma and Embryonal Carcinoma Footprints Identified by Analysis of Integrated Genome-Wide Epigenetic and Expression Profiles of Germ Cell Cancer Cell Lines
Source: PLoS One. 2014 Jun 2;9(6):e98330. doi: 10.1371/journal.pone.0098330 (PMC4041891; doi:10.1371/journal.pone.0098330)
Supplement: File S1 — ZIP file containing DMRforPairs output for significant regions. Please start from the html files. (ZIP) [file pone.0098330.s008.zip › figures/28998.pdf]

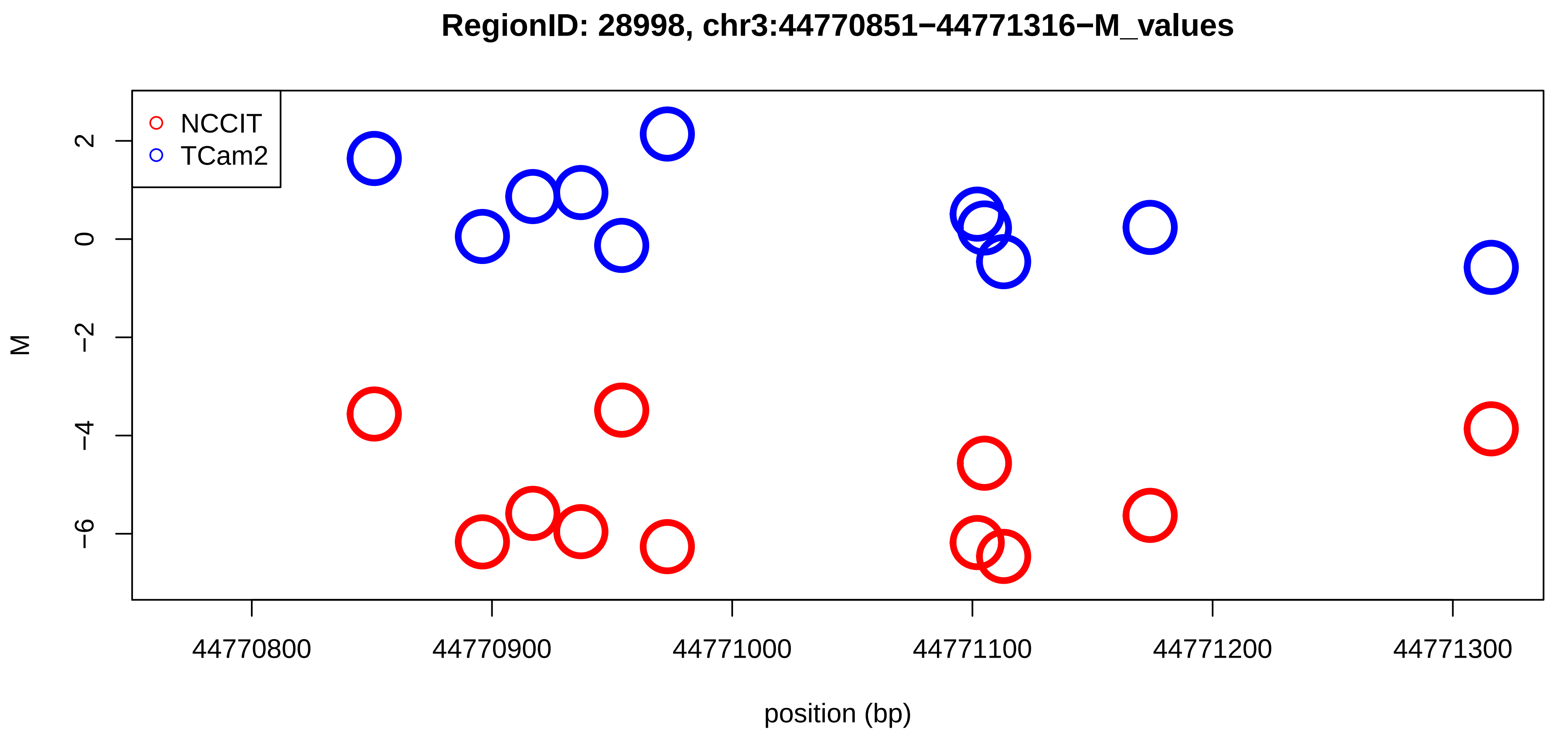

RegionID: 28998, chr3:44770851−44771316−M_values
NCCIT
TCam2
M
2
0
−2
−4
−6
44770800
44770900
44771000
44771100
44771200
44771300
position (bp)

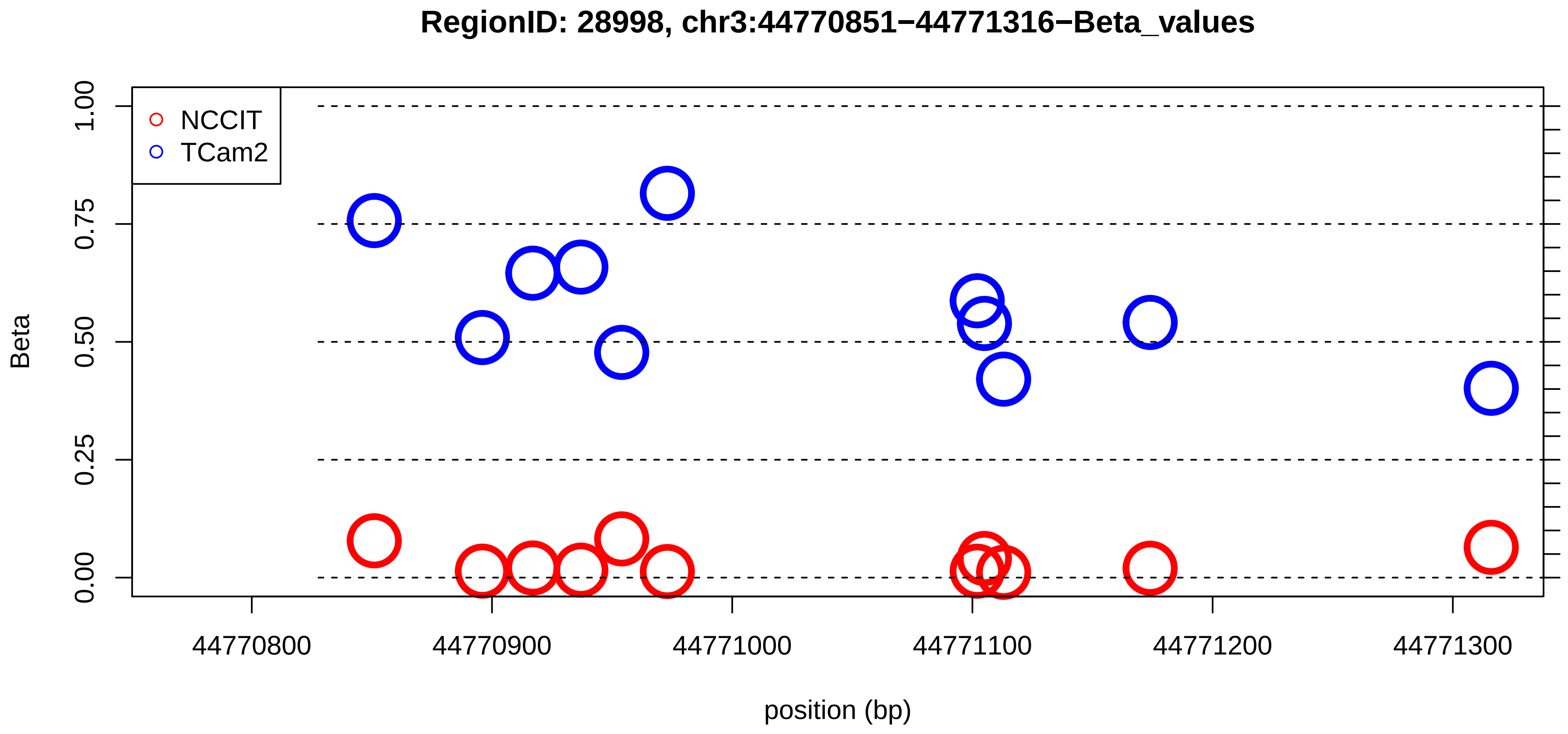

RegionID: 28998, chr3:44770851−44771316−Beta_values
NCCIT
TCam2
Beta
1.00
0.75
0.50
0.25
0.00
44770800
44770900
44771000
44771100
44771200
44771300
position (bp)

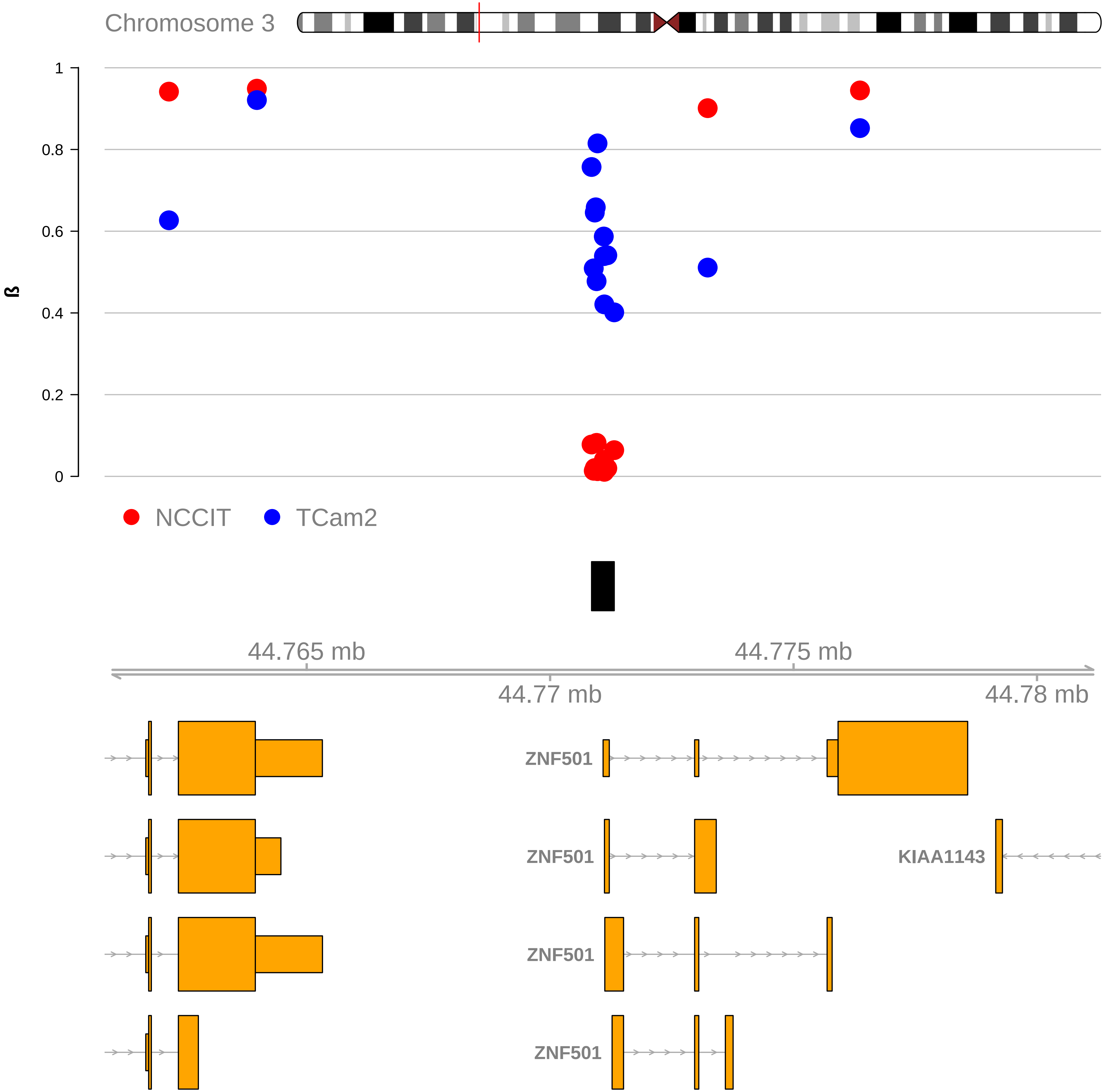
Chromosome 3
1
0.8
0.6
0.4
0.2
0
ß
NCCIT
TCam2
44.765 mb
44.775 mb
44.77 mb
44.78 mb
ZNF501
ZNF501
KIAA1143
ZNF501
ZNF501
